# Usability and Feasibility Testing of an Atrial Fibrillation Educational Website with Patients Referred to an Atrial Fibrillation Specialty Clinic

**DOI:** 10.3390/ijerph20186792

**Published:** 2023-09-21

**Authors:** Kathy L. Rush, Lindsay Burton, Cherisse L. Seaton, Peter Loewen, Brian P. O’Connor, Kendra Corman, Robyn Phillips, Lana Moroz, Jason G. Andrade

**Affiliations:** 1 School of Nursing, University of British Columbia-Okanagan, Kelowna, BC V1V 1V7, Canadacherisse.seaton@ubc.ca (C.L.S.);; 2 Department of Medicine, University of British Columbia, Vancouver, BC V1V 1V7, Canada; peter.loewen@ubc.ca (P.L.); jason.andrade@vch.ca (J.G.A.); 3 Faculty of Pharmaceutical Sciences, University of British Columbia, Vancouver, BC V1V 1V7, Canada; 4 Department of Psychology, University of British Columbia-Okanagan, Kelowna, BC V1V 1V7, Canada; 5 Cardiac Atrial Fibrillation Specialty Clinic, Vancouver General Hospital, Vancouver, BC V1V 1V7, Canada

**Keywords:** health services, atrial fibrillation clinic, website, education, user-centered design, healthcare improvement

## Abstract

Background: The purpose of this study was to design, usability test, and explore the feasibility of a web-based educational platform/intervention for patients with atrial fibrillation (AF) as part of their virtual AF care. Methods: Participants were patients attending a specialized AF clinic. The multiple mixed-methods design included website design, think-aloud usability test, 1-month unstructured pre-testing analysis using Google Analytics, follow-up interviews, and a non-randomized one-group feasibility test using pre/post online surveys and Google Analytics. Results: Usability testing participants (*n* = 2) guided adjustments for improving navigation. Pre-testing participants’ (*n* = 9) website activity averaged four sessions (SD = 2.6) at 10 (SD 8) minutes per session during a 1-month study period. In the feasibility test, 30 patients referred to AF specialty clinic care completed the baseline survey, and 20 of these completed the 6-month follow-up survey. A total of 19 patients accessed the website over the 6 months, and all 30 participants were sent email prompts containing information from the website. Health-related quality of life, treatment satisfaction, household activity, and AF knowledge scores were higher at follow-up than baseline. There was an overall downward trend in self-reported healthcare utilization at follow-up. Conclusions: Access to a credible education website for patients with AF has great potential to complement virtual and hybrid models of care.

## 1. Introduction

Atrial fibrillation (AF) is the most common form of chronic arrhythmia, whose global prevalence has doubled since the early 2000s [1]. Rates of AF steadily increase with age, from 0.16% in those younger than 49 years, 4.2% in those 60–70 years, and up to 17% in those aged 80 and older [2]. Consequences of poorly managed AF include stroke, heart failure, high rates of hospitalization, emergency department (ED) use, and increased healthcare costs [3,4].

AF is challenging for both patients and healthcare providers to manage [4,5]. The shift to virtual care with the pandemic has heightened some of these challenges. In a recent qualitative study, a number of AF patients found virtual AF care to be sub-optimal for meeting their informational and educational needs, particularly when providers communicated more complex and technical information and without the use of visuals to supplement their explanations [6]. Additionally, fewer instances of information given by both clinicians and patients were found between videoconference and phone compared to face-to-face in a UK study of primary care [7].

Evidence indicates that patients with AF with knowledge gaps and unmet educational needs will seek their information from other sources. In one study, rural patients with AF resorted to the use of a range of sources to meet their informational needs, such as popular magazines (e.g., Reader’s Digest, senior’s magazines), other patients with AF, or the internet [8]. Further, while some had uncertainties about the reliability of the information, having received warnings from health providers about internet information credibility, others accepted the information unquestioningly [8]. Similarly, Redman et al. [9] found that AF patients with important unmet informational needs may seek “unproven” advice from the internet. In contrast, Salmasi et al. [10] found that urban-based patients experienced limitations when using the internet to find information on AF, such as finding biases and inaccurate, misleading, and conflicting information.

Online/Web-based resources have shown considerable benefits for patient populations in accessing information. However, evidence suggests that the quality of online resources specific for patients with AF may not be optimal [11,12]. Pandya et al. [12] qualitatively assessed the information provided in a range of online resources to identify what aspects of thromboprophylaxis (antithrombotic treatment options) were most commonly described in these resources. They reported that the resources had suboptimal information quality. Similarly, Cano Valls et al.’s [13] assessment of websites describing catheter ablation treatment for atrial fibrillation found few resources that were of high quality, understandable, and contained actionable information. Access to and use of quality, credible, user-friendly education websites for patients with AF has great potential to complement virtual and hybrid models of care. The integration of complementary resources into the routine delivery of these care models can also support their sustainability [14]. Therefore, this study aimed to design, usability test, and explore the feasibility of a web-based educational platform/intervention for AF patients as an adjunct to their virtual AF care.

## 2. Materials and Methods

### 2.1. Study Design and Context

This study used multiple mixed methods, including qualitative website usability testing, a combination of qualitative (interviews) and quantitative (usage) approaches for website pre-testing, and a one-group pre-post educational intervention to explore the feasibility. The context was an urban-based specialized AF clinic providing in-person and virtual care to patients throughout a Canadian province. The study involved two phases: health technology intervention design/usability testing and feasibility testing. The intervention was a web-based education program combined with monthly email prompts within the context of primarily specialty virtual AF care with in-person appointments as needed.

### 2.2. Health Technology Intervention Development and Description

A dedicated AF educational website was purposefully designed to complement the education provided during specialty AF clinic appointments. The website was developed by a team of researchers and clinicians with support from information technologists and a digital marketing strategist. It used iterative design principles and proceeded through four iterations—initial content creation, webpage design, usability testing, and pre-testing before exploring feasibility.

#### 2.2.1. Content Creation

The website content included well-established knowledge/educational topics needed by patients living with and managing AF: (1) What is AF, (2) Lifestyle, (3) Complications, (4) Risk Factors, and (5) Management [15]. The content was developed using current evidence-based AF information sources (e.g., meta-analyses, systematic reviews, websites (e.g., Heart and Stroke Foundation), a patient/provider needs assessment [8]) and was guided by a multi-disciplinary group of content experts. The website content included responses prepared by clinicians (nurse practitioner (NP), cardiologist) to Frequently Asked Questions (FAQs) that arose from a previous needs assessment [8]. Selected patient stories from an earlier AF patient journey study were also integrated into the website [16]. Clinic care providers (electrophysiologist/cardiologist, NP, RN, pharmacist) vetted and validated all content.

#### 2.2.2. Website Design

**Figure 1:**
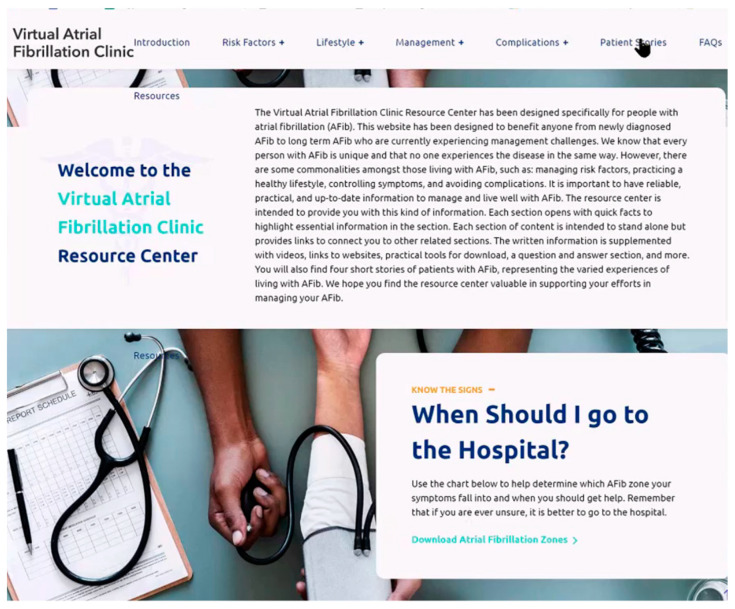
Website home page screenshot.

**Figure 2:**
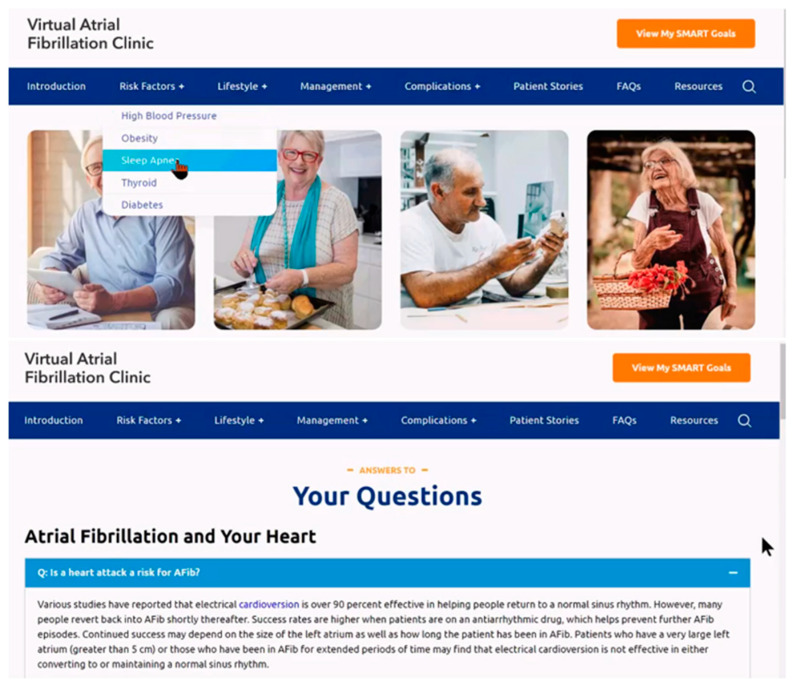
Patient stories and FAQ webpage.

A website designer built page skeletons with color schemes and font options. Research team members vetted the website structure and scheme before the content was added. Once the website was constructed, team members and AF healthcare providers were provided with a website link and asked to provide feedback on the content, readability, ease of use, and missing or extraneous information. See Figure 1; Figure 2 for website screenshots.

#### 2.2.3. Usability Testing

Two AF patients were recruited to perform usability testing from a pool of previous research participants expressing an interest in future research. The think-aloud approach, common for evaluating website usability [17,18,19], was used to have participants navigate five common website tasks while verbalizing their process. Following task completion, they were asked semi-structured questions about the website to address three criteria: (1) ease of finding information (i.e., was information where you expected it); (2) ease in understanding content; and (3) clarity of layout to support task performance. Participants found the website useful, as one participant expressed, “To me this is user-friendly” and “I’m really impressed with this because everything that you are wondering about, the answers are right here” (Male, 81 years old). Overall improvements to readability and site navigation were made based on participants’ feedback.

#### 2.2.4. Pre-Testing

Nine patients (6 males, 3 females, mean age of 53 [SD 16.1]) with new or established AF and not affiliated with a specialty AF clinic participated in a one-month unstructured pre-testing phase to evaluate their interactions with the website. They received an email giving them a link to the website and were told they had access to unlimited use of the website for 4 weeks. Google Analytics [20] was used to collect data about total time spent on the website, total number of sessions, pageviews, events, and average time spent on the website per session. Participants’ website activity averaged four sessions (SD 2.6) at 10 (SD 8) minutes per session for 42 (SD 38) minutes in total during the 1-month study period. The pages viewed most often were Lifestyle, Complications of AF, and Patient stories, followed by Medications, each with seven users.

After the 1-month period, participants were invited to participate in a follow-up interview guided by the Perceived Health Web Site Usability Questionnaire [21]. Of the nine, three participants (male 58, female 68, male 84) agreed to participate in the follow-up interviews. They found the website valuable as a refresher with everything in one place, as complementary to provider education, and as an impetus for self-reflection. They described the “good/very good” quality of the website information, its precision, simplicity, layout, and ease in understanding and singled out specific content they appreciated (e.g., risk factors). They also found it user-friendly and easy to navigate.

Beyond copyediting, participants had suggestions for website changes. These included translating it to French and adding a mechanism to encourage ongoing connection to the website (e.g., blog, educational newsletter for AF updates/self-management tips). Feedback was used to create a series of email prompts to promote website engagement.

### 2.3. Health Technology Intervention

#### 2.3.1. Design

The study used a non-randomized pre/post one-group design.

#### 2.3.2. Study Population and Setting

The feasibility study took place in a Western Canadian urban-based specialized AF clinic. The provincial-wide referral center provides integrated, multi-disciplinary care, including acute interventions, education, disease management, and advanced treatments. The clinic converted to primarily telephone appointments at the onset of the COVID-19 pandemic, with occasional video or in-person appointments as necessary. Educational sessions previously held in person were not available during the pandemic nor at the time of publication. Therefore, the website aimed to fill educational gaps and complement specialty care appointments.

Eligible participants were any newly referred patients to the AF clinic who had not yet had their first appointment. Such patients were typically recently diagnosed, symptomatic, and/or referred for advanced treatment (e.g., ablation). Patients received a letter (email or mail) notification from the clinic booking clerk about the research study and the potential for research team contact with them regarding participation. During recruitment, Research Assistants (nursing or medical students) recruited patients using the clinic schedule with patient contact information and appointment types. They identified and telephoned eligible patients to provide study details and answer any outstanding questions. Recruitment began in January 2021 and continued until September 2021, after which recruitment was closed due to project timelines and the 6-month follow-up. Due to recruitment challenges, including challenges contacting/reaching eligible patients prior to their first appointment, participant numbers remained low, and we were unable to recruit sufficient numbers for a control group.

#### 2.3.3. Intervention Procedure

Following consent and baseline measure completion, all participants received an intervention welcome email, instructions on website access, and general information about the website. Participants were instructed to visit the website at their convenience and to expect regular emails from the research team. Website usage was unstructured to allow participants to control when they used the website, for how long, and what content they accessed. An unstructured web-based educational approach has been previously shown to improve self-care [22]. Website interaction was encouraged by monthly email prompts (*n* = 6) sent to participants during the intervention. These encouraged making lifestyle changes with SMART goals, integrating exercise, when to seek healthcare, controlling risk factors, healthcare provider communication, and healthy diet.

#### 2.3.4. Data Collection

Data were collected using an online survey hosted on Qualtrics [23] and Google Analytics [20]. Patients needing additional assistance with survey completion could do it over the phone with an RA or with a family member/friend.

#### 2.3.5. Measures

*Socio-demographic characteristics*. Questions asked about age, sex, marital status, race/ethnicity, education, and income.

*Technology*. Researcher-developed questions asked participants to select the types of technology they used for daily life and healthcare (e.g., appointments, information) as well as the type and cost of internet service. The questions also asked participants to rate their satisfaction with internet services on a scale from 1 (poor) to 10 (excellent) on reliability, speed, support, security, and availability.

*Self-Efficacy and Healthcare Technology Attitudes* [24]. A validated 24-item tool that captures general self-efficacy, health technology self-efficacy (HTSE), and attitude toward healthcare technology. Items are scored on a seven-point Likert scale from 1 (strongly disagree) to 7 (strongly agree). Scores range from 1 (low self-efficacy/attitude) to 7 (high self-efficacy/positive attitude).

*Overall Health.* Participants were asked to rate their overall health on a scale from 1 (poor) to 4 (excellent) [25].

*Overall mental health*. Participants were asked to rate their overall mental health on a scale from 1 (poor) to 4 (excellent) [26].

*Perceived stress* [27]. The Perceived Stress Scale (PSS-10), a validated 10-item, 5-point scale, measures the degree to which situations in one’s life are appraised as stressful, the ability to control aspects of life, confidence in handling problems, or being unable to cope with demands.

*AF Knowledge* [15]. The validated knowledge about the AF tool is a 24-item multiple choice-style questionnaire, including questions about AF symptoms, treatment, medications, risk factors, and lifestyle. Participants are asked to choose one of three options for each question, only one of which is the correct response. Knowledge scores are calculated as a percentage of correct answers, with higher numbers indicating higher knowledge.

*Household and recreational physical activity* [28]. Physical activity scores were calculated based on the validated Phone-FITT questionnaire, adapted for online administration. Participants were asked to indicate their participation in various household and recreational activities in a typical week within the past month. Participants were also asked to provide a frequency (times per week) and choose a duration from 1 (1–15 min) to 4 (1 h or more) and an intensity from 1 (breathing normally and able to carry on a conversation) to 3 (too out of breath to carry on a conversation). Scores were calculated as the sum of the frequency, duration, and intensity for all household and recreational activities, with higher scores indicating higher physical activity.

*Atrial Fibrillation Effect on QualiTy-of-Life Questionnaire* (AFEQT) [29]. AFEQT is a 20-item, 7-point scale comprising overall HRQoL and 3 sub-domains: symptoms, daily activities, treatment concerns, along with AF treatment satisfaction. Overall HRQoL scores are calculated as the sum of items 1–18, accounting for unanswered items, and normed on a scale from 0–100, with higher numbers indicating higher HRQoL. Treatment satisfaction scores for items 19–20 follow the same calculation, with higher scores indicating higher treatment satisfaction.

*Healthcare Utilization*. A researcher developed a self-reporting tool to capture healthcare utilization in the past six months, including the number of AF-related appointments per healthcare provider, appointment type (i.e., in-person, virtual), emergency department visits, and hospitalizations. Self-reported healthcare utilization has shown good agreement with administrative data [30].

#### 2.3.6. Analysis

Website usage was analyzed using Google Analytics to measure the total time spent on the website, the total number of website sessions, the total number of page views, the total number of events, and the average time each participant spent on the website per session. Google Analytics was also used to track what device participants were using to access the website (e.g., computer or smartphone) and whether directly (i.e., typing the website address) or following a researcher-provided link (e.g., welcome email, newsletter link).

Data were analyzed using SPSS Statistics 28 [31]. Descriptive analyses (e.g., means and frequencies) were used to summarize the data. To evaluate the differences in all outcome variables of participants completing both baseline and the post-intervention 6-month follow-up surveys, paired samples *t*-tests were used. The magnitude of the pre- and post-intervention differences was evaluated based on the effect size (Cohen’s D), given the small sample size [32,33]. All continuous variables were inspected for normality and outliers. *p*-values are used solely for informational/descriptive purposes, not to infer the effects of the intervention.

### 2.4. Ethical Considerations

University Behavioral Research Ethics Board approval was obtained (#H19-03601) prior to data collection. This study has been reported in line with the Consolidated Standards of Reporting Trials (CONSORT) extension for reports of randomized pilot and feasibility studies [34].

## 3. Results

Of an original panel of 198 new referrals during the feasibility study period, 54 became ineligible (either participated in another related study or were not reached prior to their first appointment). Of the remaining 144, 30 participants (20.8% response rate) completed the baseline survey, and 20 of these completed the 6-month follow-up survey (approximately 33% lost to follow-up). Baseline surveys took on average 44 min (range 19–89) to complete, and follow-up surveys took on average 38 min (range 15–79). Baseline surveys had 1.8% missing data, with no consistent pattern for missingness (Little’s MCAR *p* = 0.742). The initial sample (*n* = 30) had an average age of 66 (range 37 to 84 years; SD = 9.8) and was primarily Caucasian (*n* = 27; 90%). Baseline characteristics of participants are presented in Table 1. Of these participants, 53% reported using either a desktop or laptop computer, 47% used a tablet, and 47% used a smartphone in their daily lives. Most participants reported having “good” or “excellent” physical health (*n* = 21; 70%) and mental health (*n* = 21; 70%), and on average perceived stress was relatively low at baseline (M = 13.93; SD = 8.1).

### 3.1. Healthcare Utilization and AF Specialty Clinic Care

At baseline (*n* = 30), the most common healthcare practitioner that participants reported visiting in relation to their AF was a GP (*n* = 18; 60%), followed by a specialist (*n* = 9; 30%), then a pharmacist (*n* = 7; 23%). The average number of GP visits for the 18 participants was 2.72 times (SD = 1.32) in the past 6 months. Of 27 participants at baseline, 43% (*n* = 13) indicated that they had visited the emergency department (ED) in the past 6 months because of their AF. Of these 13 participants, 2 reported being hospitalized once after visiting the ED, and 3 reported being hospitalized 3 times. On average, these 5 participants were hospitalized for about 2.6 days (SD = 1.34).

At the 6-month follow-up (*n* = 20), 8 participants reported visiting their GP (40%), 3 visited a specialist (15%), and 5 visited a pharmacist (17%) in relation to their AF. The average number of GP visits for these 8 participants was 2.63 times (SD = 1.19). Of the 20 who responded, only 1 indicated that they had visited the ED in the past 6 months for their AF, and this participant did not report being hospitalized.

For the 20 patients who completed the follow-up survey, the average number of AF clinic encounters over the 6 months was 7.70 (SD = 6.47). The average number of encounter appointments for those who completed the follow-up and used the website (N = 15) was 8.73 (SD = 7.12) and 4.60 (SD = 2.41) for those who completed the follow-up and did not use the website (N = 5). 

### 3.2. Website Usage

Table 2 compares the website usage of all participants who accessed the website, patients who accessed the website and completed both pre and post surveys, and patients who accessed the website but completed the baseline survey only.

Of the 30 participants who completed the baseline measures and were given access to the website, 19 accessed the website (15 of these completed follow-up measures, and 4 did not) over the 6 months either from a computer (*n* = 15), a smartphone (*n* = 3), or a tablet (*n* = 1). The mean number of website sessions was 2.5 (SD 1.57; Range: 1 to 7 sessions), with a mean time per session of 8.6 min (SD = 11.1). There were 68 unique webpage visits between the 19 participants during the 6 months.

All 30 participants were sent email prompts that contained information about AF drawn from the website. Most participants (*n* = 18) accessed the website from a link emailed to the participants, with one participant accessing the website by typing or copying the address into the browser directly. Participants accessed the website using links from the introductory (*n* = 11), month one lifestyle (*n* = 10), month two exercise (*n* = 2), and month four risk factor emails (*n* = 8). Participants did not access the website by following email links from months 3 (symptom management), 5 (health provider communication), and 6 (healthy diet). Participants primarily visited the home page (53% of visits/sessions), with the risk factors (20% of visits/sessions) and lifestyle (18% of visits/sessions) pages the next most commonly visited pages. The time from researcher communication to the website visit was, on average, 1.8 days, with approximately three participants visiting the website on the same day of email receipt. Webpages that did not have specific prompts—patient stories, resources, and complications—received fewer visits.

### 3.3. Participants at Baseline versus Follow-Up

The descriptive data and results of paired samples *t*-test for participants at baseline and at follow-up are presented in Table 3.

Table 3 shows the means and mean change scores, confidence intervals, and effect sizes for all study variables from baseline to follow-up. Overall, the intervention (virtual care/AF website and prompts) produced mixed effects—positive, negative, and no change. The largest changes were observed in variables showing positive effects: HRQoL, treatment satisfaction, and household activity. The mean HRQoL, treatment satisfaction, and household activity scores were higher at follow-up than baseline, with medium to high effect.

A smaller positive effect was observed for change in AF knowledge. The average baseline knowledge score was higher at follow-up, with a small effect (d = 0.29). The knowledge sub-scale mean scores that were higher at follow-up were risk factors knowledge, basic AF, and common symptom knowledge.

Two variables displayed small negative effects. These included lower recreational activity at follow-up (d = 0.20, small effect) and higher perceived stress at follow-up (d = 0.26, small effect).

## 4. Discussion

This study aimed to design, usability test, and explore the feasibility of a web-based educational platform/intervention for AF patients to complement the virtual care and education provided at an urban-based specialty AF care clinic. Following 6-month access to and use of a dedicated AF website together with virtual care sessions, we observed increases in HRQoL, treatment satisfaction, and household activity for patients attending the clinic. AF knowledge was also higher at follow-up, although the effect did not reach significance.

The recruitment rate (20.8%) overall was fairly low, though retention (67%) was reasonable. According to the framework for hypothesis testing and sample size around process outcome evaluation, future pilot study work would require a minimum sample of 78 to verify/confirm a minimum recruitment uptake of 20% [35]. Our findings suggest that to accomplish this, either the recruitment timeframe would need to be extended or recruitment occurs through more than one clinic; however, establishing avenues to bolster recruitment, such as reducing participant burden or increasing incentives to participate, would enhance feasibility. However, our recruitment and retention were consistent with other pre-post interventions with AF patient populations, which have ranged from as low as 13.2% of eligible patients with AF presenting for consultation or hospitalized [36] to 65% of patients undergoing pulmonary vein isolation or cardioversion recruited to use an online education platform [37]. Given recruitment rates are often unreported in studies of patients with AF, our findings provide useful uptake estimates among patients with AF newly referred to a specialty clinic. Finally, despite fairly lengthy online survey completion times (~40 min), we did not identify high levels of missing data or critical issues with the included study measures, though the follow-up survey completion rate may have been higher had surveys been shorter/less burdensome.

As recommended for feasibility studies, we did not perform formal hypothesis testing for intervention effectiveness but instead examined the magnitude of effect [38]. Following Cohen’s [39] guide for effect sizes (d = 0.2 = small, d = 0.5 = medium, and d = 0.8 = large), we found a large effect size for HRQoL (d = 0.88) and medium effect sizes for treatment satisfaction (d = 0.56) and household activity (d = 0.51), suggesting that these differences were quite substantial. These findings resonate with findings from a recent study of a nurse-led primary care clinic in a Singapore community that offered integrated chronic care, including AF with Advanced Practice nurse delivered regular patient education, supplemented by a specially curated webpage, fast-tracked appointments for hospital-based specialized investigations, and teleconsultation with a hospital-based cardiologist [40]. These authors reported increased AF knowledge, HRQoL, medication adherence, patient satisfaction, and improved depression from baseline to 6-month follow-up in their one group pre-post intervention study but no change in cardiovascular-related hospitalizations. Similarly, a systematic review of seven studies evaluating the effectiveness of different apps for AF management reported that overall these improved patient knowledge of AF, HRQoL, and medication adherence [41].

The nearly 20-point increase in HRQoL from pre- to post-intervention among patient participants in the current study is well above the 5-point change in HRQoL that is considered meaningful [42]. This increase is reflected in increases in the symptoms, daily activities, and treatment concerns subscales. Symptoms have been well-established to be related to HRQoL [43]. This trend is consistent with participants’ modest increases in treatment satisfaction and household activities and in the overall downward trend in ED visits observed over the 6-month intervention. The observed increase in HRQoL and household activity is consistent with our other work in which we found that higher household physical activity, but not recreational activity, was related to higher HRQoL on all three subscales, as well as overall HRQoL [44]. Study patients demonstrated higher overall mean AF knowledge scores at 6-month follow-up that represented a small but non-significant effect size. The overall total baseline knowledge scores were average at 79 (M 18.89/24 SD 3.38) and increased to 83 (M 19.75/24 SD 2.10) post-intervention. This small 3-point increase may reflect the variability across the knowledge sub-domains from a low of 60 on consequences of knowledge to 97 on common symptom knowledge. This is not unexpected given that participants, despite all being “new referrals”, included both patients with newly diagnosed AF and with longer-standing AF receiving specialized treatments/management (e.g., ablation). Higher scores might be expected for those with longer standing AF compared to those recently diagnosed. This is consistent with McCabe et al.’s [15] initial psychometric testing of their Knowledge about AF tool with higher scores in patients undergoing ablation compared to newly diagnosed patients.

The small rise in total knowledge scores reflects sub-domain score increases in those sub-domains with the highest baseline scores. Although it is not possible to determine the complementary contribution of the website to the observed effects separate from the virtual clinic appointments and supports, these specific sub-domain increases align with both the greater number of visits to webpages, which included these three knowledge domains (home page: basic AF and symptom management; and risk factors page) and email prompts that triggered participants to visit webpages, including these knowledge domains. In another study, a virtual intervention increased knowledge beyond information available during the often brief clinic visits [45]. Noteworthy was the minimal change and, in some cases, decrease in scores of those sub-domains on which participants scored the lowest at baseline (e.g., consequences, treatment, monitoring). Engaging patients in these important sub-domains should be an emphasis in future web-based work with AF patients.

Homepage visits garnered the greatest proportion of patients’ website visits, and although not unexpected, it points to the importance of including critical, need-to-know information on this page. The AF website homepage used in the current study included a website overview, what AF is (plus video), AF types and symptoms, and downloadable guidelines for when to go to the hospital. Risk factor pages had the next highest visits and reinforced the appreciation pre-testing participants expressed related to this content. Lifestyle webpages, the third most visited pages, are an important finding. A strength of the AF website used in the current study is its inclusion of pages related to lifestyle factors (e.g., obesity, alcohol, smoking, physical activity), content not always found on publicly available AF websites. In fact, in their systematic review of AF websites, Middledorp et al. [46] found that only 74% of the websites provided information on lifestyle factors associated with AF, while AF definition, symptoms, types, medication therapies, and procedural treatment options appeared in over 90% of them. That patients appeared to gravitate to the risk factor and lifestyle pages may also reflect their interest in learning ways to self-manage their AF beyond treatments.

The use of a largely unstructured web-based educational approach, previously shown to improve self-care [22], together with email prompts to trigger webpage viewing in the current study yielded website engagement of nearly two-thirds of participants during the 6 months. The email prompts, in particular, appeared to promote website engagement, mostly within two days of email receipt and many the same day. This enhanced engagement is consistent with the findings from a systematic review that reported technology-based prompts had borderline positive effects compared to no prompts [47]. Although website users had nearly double the clinic appointments of non-users, whether appointments with clinic providers played any role in website engagement is unknown but bears further exploration. Noteworthy was our finding that website use on pages not highlighted in emails had little or no views. Overall, an educational website was feasible for complementing the use of virtual care to meet the educational needs of patients with AF. Expanding support for patients with AF, as in the current study, enhances the quality and efficiency of hybrid and virtual models of care and has the potential to advance their sustainability.

### Limitations

This study provides valuable foundational knowledge for expanding and evaluating an educational website to support AF patients as part of their AF specialty clinic care. Despite its strengths, this study has some limitations. The small sample size, which aligns with our objective of assessing feasibility, limits the generalizability of our findings, as does the specific focus on patients newly referred to AF specialty clinic care. Although the largely Caucasian sample reflected the larger clinic population of patients with AF, it lacked representation of ethnic diversity. The pre-post findings should be interpreted cautiously, as the estimation of treatment effects in feasibility studies can be unreliable due to small sample sizes [48]. Yet, according to Sim [38], confidence intervals around mean differences can provide informal reassurance of what might be expected in a future study. Indeed, for overall HRQoL, the lower bound 95% confidence interval was above the minimally important change (MIC) from a clinical perspective (e.g., 5-point difference [42]), suggesting that an effect of at least the MIC will be observed in similar future intervention research. We lost one-third of the participants in the follow-up and some participants did not access the website at all. The lack of a control group limits the strength of conclusions we can draw about the contribution of the website. Despite this, changes in knowledge sub-domains did align with most accessed website content. Although there was no tracking of the “education” participants received as part of their virtual appointments or additional education outside of the clinic during the 6-month study, it is likely that primary care physicians, who were the most frequent health provider participants, saw during the study period, may have provided education. Additionally, all participants were provided email prompts with similar content to the online web pages, which limits the interpretability of comparing website users and non-users. Future research might include testing the website and specialty care clinic against care alone as an active control group.

## 5. Conclusions

Newly referred patients to AF specialty clinic care who received specialty AF care and website access with email prompts over 6 months had higher scores on health-related quality of life, treatment satisfaction, household activity, and AF knowledge compared to baseline. They engaged mainly with the home, lifestyle, and risk factor pages, and email prompts encouraged engagement. These feasibility findings may provide a foundation to inform the design and conduct of future pilot trials on patients with AF. Although further testing is needed, there is considerable potential for eHealth and educational interventions to complement virtual and hybrid models of healthcare and enhance their sustainability.

## Figures and Tables

**Table 1:** Characteristics of participants at baseline.

Characteristics	All Participants (*n* = 30)	Participants Who Completed Follow-Up (*n* = 20)
**Age ^1^**	**66 (10)**	**65 (10)**
**Sex ^2^**		
Female	13 (43)	9 (45)
Male	17 (57)	11 (55)
**Ethnicity ^2^**		
Caucasian/White	27 (90)	17 (85)
Asian	2 (6.7)	2 (10)
Indigenous	1 (3.3)	1 (5.0)
**Marital Status ^2^**		
Single, divorced, separated, or widowed	13 (43)	8 (40)
Married, remarried, or common law	17 (57)	12 (60)
**Education ^2^**		
College/University Graduate	17 (57)	14 (70)
Some post-secondary	7 (23)	4 (20)
High School or less	6 (20)	2 (10)
**Income ^2^**		
Less than $25,000	6 (20)	2 (10)
$25,000–$50,000	8 (27)	6 (30)
$51,000–$75,000	4 (13)	1 (5)
Over $75,000	12 (40)	11 (55)
**Overall Health ^2^**		
Excellent	2 (6.7)	2 (10)
Good	19 (63)	14 (70)
Fair	8 (27)	4 (20)
Poor	1 (3.3)	0
**Overall Mental Health ^2^**		
Excellent	7 (23)	7 (35)
Good	14 (47)	8 (40)
Fair	7 (23)	4 (20)
Poor	2 (6.7)	1 (5)
**Last Atrial Fibrillation ^2^**		
Current	10 (33)	7 (35)
Earlier today	2 (6.7)	1 (5)
Within the past week	5 (17)	3 (15)
Within the past month	6 (20)	4 (20)
1 month to 1 year ago	5 (17)	4 (20)
Never aware	1 (3.3)	1 (5)
**Tobacco Use ^2^**		
Yes	2 (6.7)	1 (5)
No	28 (93)	19 (95)
**Alcohol Use ^2^**		
Yes	12 (40)	10 (50)
No	18 (60)	10 (50)
**Fruit consumption (days per week) ^2^**		
1–2	3 (10)	1 (5)
3–4	4 (13)	2 (10)
5–6	7 (23)	5 (25)
>6	16 (53)	12 (60)
**Servings of fruit per day ^2^**		
1–2	21 (70)	13 (65)
3–4	8 (27)	7 (35)
5–6	1 (3.3)	0
**Vegetable consumption (days per week) ^2^**		
1–2	2 (6.7)	2 (10)
2–3	1 (3.3)	0
3–4	2 (6.7)	1 (5)
5–6	9 (30)	7 (35)
>6	15 (50)	9 (47)
Missing	1	1
**Servings of vegetables per day ^2^**		
1–2	11 (37)	6 (30)
3–4	16 (53)	13 (65)
4–5	1 (3.3)	0
5–6	1 (3.3)	1 (5)
>6	1 (3.3)	0
**Cooking fat used ^2^**		
Vegetable oil	24 (80)	16 (80)
Butter	3 (10)	1 (5)
Margarine	2 (6.7)	2 (10)
Missing	1	1

^1^ Mean (SD). ^2^ *n* (%).

**Table 2:** Comparison of Website Users.

	Total (*n* = 19)	Pre/Post (*n* = 13)	Pre (*n* = 6)
**Average # of unique pages visited ^a^**	3.58 (1.95)	3.92 (2.13)	2.83 (1.32)
**Average # of sessions ^a^**	2.52 (1.57)	2.85 (1.62)	1.83 (1.33)
**Email Prompts ^b^**			
None	1	/	1
Introduction	8	6	2
Lifestyle (1)	8	6	2
Exercise (2)	2	2	
Risk Factors (4)	7	4	3
**Unique Pages Visited ^b^**			
Home Page	12	9	3
Lifestyle	13	11	2
Risk Factors	10	7	3
Symptom Management	8	7	1
Complications	4	3	1
FAQ	7	6	1
Patient Stories	2	/	2
Resources	6	4	2
Smart Goals	6	4	2
**Acquisition ^b^**			
Introductory Email	11	9	2
Lifestyle Campaign (First Email Prompt)	3	2	1
Risk Factor Campaign (4th email prompt)	4	2	2
Typed in website directly (no link followed)	1	/	1

^a^ mean (SD). ^b^ participant counts. # = number

**Table 3:** Baseline and 6-month follow-up comparisons.

Variable	Participants at Baseline (*n* = 20) ^1^	Participants at Follow-Up (*n* = 20) ^1^	Change M (95% CI)	t	*p*	Cohen’s D (95% CI)
AF Knowledge Missing *n* = 2	78.70 (14.07)	81.71 (8.94)	3.01 (−2.11, 8.12)	1.24	0.231	**0.29** (−0.18, 0.76)
AF Subscales:						
Basic AF Knowledge	90.74 (19.15)	100.0 (0.0)	9.26 (−0.26, 18.78)	2.05	0.056	**0.48** (−0.01, 0.97)
Common symptom knowledge	97.22 (11.79)	100.0 (0.0)	2.78 (−3.08, 8.64)	1.00	0.331	**0.24** (−0.24, 0.70)
Consequences knowledge	60.0 (22.67)	61.33 (24.46)	1.33 (−10.84, 13.51)	0.24	0.818	0.06 (−0.45, 0.57)
Recurrent knowledge	82.35 (20.81)	78.43 (23.40)	−3.92 (−18.62, 10.77)	−0.57	0.579	−0.14 (−0.61, 0.34)
Treatment Knowledge	70.0 (28.92)	70.0 (20.00)	0.0 −13.27, 13.27)	0.00	1.00	0.0 (−0.57, 0.57)
Monitoring knowledge	83.33 (11.79)	84.31 (13.78)	0.98 (−4.66, 6.62)	0.37	0.718	0.09 (−0.39, 0.56)
Risk factors knowledge	94.12 (13.10)	100 (0.0)	5.88 (−0.85, 12.62)	1.85	0.083	**0.45** (−0.06, 0.94)
Psyc knowledge	94.44 (23.57)	94.44 (23.57)	0.0 (−17.06, 17.06)	0.00	1.00	0.00 (−0.46, 0.46)
Household Activity Missing *n* = 1	25.84 (6.67)	29.58 (5.98)	3.74 (0.20, 7.28)	2.22	0.040	**0.51** (0.02, 0.98)
Recreational Activity Missing *n* = 1	28.13 (18.67)	25.77 (13.35)	−2.36 (−7.94, 3.23)	−0.89	0.388	**−0.20** (−0.66, 0.25)
Overall HRQoL (AFEQT)	68.72 (22.23)	84.28 (11.82)	15.56 (7.31, 23.80)	3.95	<0.001	**0.88** (0.36, 1.40)
Symptoms HRQoL Subscale	69.17 (20.92)	86.67 (12.87)	17.5 (8.19, 26.81)	3.93	<0.001	**0.88** (0.35, 1.39)
Daily Activities Subscale	73.41 (23.57)	84.91 (15.44)	11.50 (2.83, 20.18)	2.77	0.012	**0.62** (0.13, 1.09)
Treatment Control Subscale	62.92 (27.58)	81.81 (14.88)	18.89 (8.74, 29.03)	3.90	<0.001	**0.87** (0.35, 1.38)
Treatment Satisfaction (AFEQT) Missing *n* = 1	60.53 (25.59)	81.14 (26.62)	20.61 (2.78, 37.44)	2.43	0.026	**0.56** (0.07, 1.04)
General SE Missing *n* = 2	6.01 (0.81)	5.90 (0.95)	−0.10 (−0.47, 0.27)	−0.59	0.564	−0.14 (−0.60, 0.33)
Health technology SE Missing n = 2	5.23 (1.14)	5.24 (1.25)	0.01 (−0.40, 0.42)	0.05	0.962	0.01 (−0.45, 0.47)
Technology attitude Missing *n* = 2	4.99 (1.09)	5.13 (1.38)	0.14 (−0.31, 0.60)	0.67	0.512	0.16 (−0.31, 0.62)
Perceived Stress	12.80 (8.12)	14.70 (8.74)	1.90 (−1.56, 5.36)	1.15	0.265	**0.26** (−0.19, 0.70)

^1^ Mean (SD). Notes: Participants who did not complete a measure at baseline or at follow-up are indicated as “Missing”. Effect sizes that are small, medium, or large in magnitude, according to Cohen (1988), are bolded regardless of whether they are significant based on formal hypothesis testing.

## Data Availability

Data are unavailable due to ethical restrictions.
